# Role of sleeve gastrectomy in improving metabolic syndrome: an overview

**DOI:** 10.1007/s13304-024-02038-4

**Published:** 2024-11-25

**Authors:** Adisa Poljo, Marko Kraljević, Ralph Peterli, Beat P. Müller, Adrian T. Billeter

**Affiliations:** Division of Metabolic Bariatric Surgery, Department of Visceral Surgery, Clarunis, University Digestive Health Care Center Basel, St. Clara Hospital and University Hospital Basel, Basel, Switzerland

**Keywords:** Gastric sleeve, Metabolic and bariatric surgery, Metabolic syndrome

## Abstract

Metabolic syndrome (MetS) presents a global health challenge characterized by cardiometabolic risk factors like central obesity, elevated blood pressure, dyslipidemia, and high fasting glucose levels. Despite lifestyle interventions and medications, the increasing prevalence of MetS calls for effective treatments. Sleeve gastrectomy (SG) has emerged as a promising intervention. This review examines the role of SG in improving MetS outcomes, drawing from a PubMed/Medline literature search. It highlights SG’s multifaceted metabolic effects, including hormonal changes and improved insulin sensitivity, contributing to improved metabolic outcomes. Additionally, SG leads to significant weight loss and effectively addresses comorbidities like hypertension, dyslipidemia, and type 2 diabetes mellitus (T2DM), with low rates of early morbidity and mortality. However, long-term studies indicate that Roux-en-Y gastric bypass (RYGB) provides more sustained weight loss and superior resolution of metabolic comorbidities, whereas SG is associated with fewer early complications and a lower risk of nutritional deficiencies. In conclusion, SG offers a valuable option for managing MetS, providing significant weight loss and comorbidity improvement. Nevertheless, potential long-term complications, such as gastroesophageal reflux disease (GERD) and suboptimal weight response, emphasize careful patient selection and monitoring.

## Introduction

Metabolic syndrome (MetS) is a medical condition characterized by a cluster of cardiometabolic risk factors like central obesity, arterial hypertension, dyslipidemia, and elevated fasting glucose levels [1]. These comorbidities contribute to it being a chronic disease that significantly reduces quality of life, increases mortality rates, and results in substantial healthcare costs [2].

Initial treatment recommendations include adopting a healthy diet, increasing physical activity, and making behavioral modifications [3]. However, weight loss achieved through these methods is usually modest, and most people tend to regain the lost weight over time. Medication like antihypertensive and lipid-lowering drugs plays a central role in preventing atherogenesis, coronary artery disease, and cerebrovascular disease. Moreover, new drugs for the treatment of obesity, including glucagon-like peptide-1 (GLP-1) and glucose-dependent insulinotropic peptide (GIP) agonists, have shown promise in significantly aiding weight loss and improving metabolic health. However, for most patients with moderate to severe type II diabetes (T2DM) and obesity, intensive medical therapy with lifestyle changes does not sufficiently reduce glycated hemoglobin levels to effectively reverse T2DM [4].

Metabolic and bariatric surgery has proven to be an effective treatment for obesity and T2DM. When compared to non-surgical interventions, it results in greater long-term improvements in weight loss outcomes and weight-associated comorbidities, regardless of the surgical procedure [5]. This success has brought surgery to the forefront in treating MetS and has led to increasing recognition of it as a highly effective treatment for T2DM [6, 7].

Sleeve gastrectomy (SG) is currently the most commonly performed primary bariatric procedure worldwide, accounting for 62.5% of cases, followed by Roux-en-Y gastric bypass (RYGB) at 28.5%. However, the preferred procedure varies by country. In the United States, 140,339 primary SG procedures were reported, representing 68.8% of cases. Conversely, RYGB is the most commonly reported primary procedure in Brazil, Venezuela, the Netherlands, Norway, Canada, Austria, Switzerland and Sweden and the One-Anastomosis Gastric Bypass (OAGB) in Israel [8].

SG was originally intended as a preparatory step for biliopancreatic diversion with duodenal switch (BPD-DS). However, due to its remarkable efficacy in achieving significant weight loss, minimal complications, great food tolerance, and brief hospitalization, it evolved to a stand-alone procedure and into the most frequent bariatric–metabolic operation worldwide [9, 10].

## Methods

A non-systematic literature search was performed in the PubMed/Medline database during April–May 2024 to identify peer-reviewed original articles on the role of SG in improving MetS. No time restrictions were applied to ensure a comprehensive review. The search encompassed English language literature, including randomized controlled trials (RCTs), retrospective studies, single and multicenter cohorts, case reports, as well as systematic reviews and meta-analyses. Articles deemed relevant were evaluated and selected by consensus among the authors for inclusion in this comprehensive narrative synthesis of previously published information.

## Pathophysiology

Originally, SG was considered a restrictive bariatric procedure as it involves laparoscopic removal of approximately 75% of the stomach. However, over time it has been revealed that the metabolic impact goes far beyond mere caloric restriction. Several hypotheses explain these metabolic effects, including changes in the secretion of hormones like ghrelin, peptide-YY (PYY), leptin, GLP-1, GIP, and cholecystokinin (CCK), a decrease in insulin resistance, and alterations in gut microbiota [11, 12]. Patients with T2DM typically have a reduced secretion of GLP-1 and GIP. Improved glucose control may be attained by a combination of two processes, duodenal exclusion and rapid distal small intestine nutrition exposure.

SG preserves the integrity of the pylorus and does not involve intestinal bypass, so significant changes in distal gut hormone release would not be expected. Surprisingly, despite not altering the passage of food through the small intestine, comparative studies of gut hormones after SG and RYGB indicate both surgeries influence GLP-1, GIP, PYY, CCK and leptin levels [13–15]. This might be because GLP-1 release is not solely triggered by direct nutrient contact with distal L cells. Another stimulus for GLP-1 secretion comes from proximal nutrient signals, such as increased CCK secretion, which is stimulated by the formation of long-chain free fatty acids [16].

It is believed that diabetes control results from the accelerated delivery of nutrients to the distal small intestine, which enhances the release of incretins and thereby improves glucose-dependent insulin production in pancreatic beta cells.

One possible reason for the rise in PYY and GLP-1 levels post SG could stem from quicker gastric emptying, leading to earlier interaction between chyme and the L cells. Scintigraphic investigations conducted within a span of 2 years after LSG exhibited swifter gastric emptying for both solid and liquid diets [17].

Another hormone, which plays an essential role in obesity and MetS, is ghrelin. It is an appetite stimulant and is produced mainly by enteroendocrine cells in the stomach. Diet-induced weight loss raises circulating ghrelin levels, but SG significantly lowers fasting ghrelin and suppresses post-meal ghrelin [18]. This reduction in serum ghrelin levels persists at a 5 year follow-up after sleeve gastrectomy [19].

## Metabolic outcomes after sleeve gastrectomy

When it comes to managing MetS and obesity, research indicates improved outcomes following SG [10] (Table 1).

**Table 1:** Summary of metabolic outcomes after sleeve gastrectomy

Metabolic outcome	Summary
Weight loss	• 25.2% TBWL after SG at 1 year, 21.0% at 2 years, and 18.8% at 5 years, compared to 31.2% after RYGB at 1 year, 29.0% at 3 years, and 25.5% at 5 years [20] • No significant difference between SG and RYGB at 5 years [21, 22], but superior %EBMIL after RYGB in merged data analysis [23]
Arterial hypertension	• Reduced in 75% after SG, 58% complete resolution within 16.9 ± 9.8 months [24] • Higher rates of antihypertensive medication discontinuation after RYGB at both 5 years (51% vs. 29% for SG) and 10 years (24% vs. 8%) [21, 25] • Cardiac remodeling observed after SG, with decreased left ventricular mass and improved diastolic function [26]
Dyslipidemia	• After 5 years, 83.1% of patients after RYGB showed improvement in dyslipidemia, compared to 62.0% after SG [27] • Complete remission of dyslipidemia in 62.3% after RYGB patients versus 42.6% after SG [22]
Diabetes	• Higher rates of achieving glycated hemoglobin level ≤ 6.0% compared to medical therapy alone at 12 months and 5 years [4, 28] • Remission rates 59.2% for RYGB and 55.9% for SG at 1 year, rising to 86.1% and 83.5% at 5 years post-surgery [29]

*%EBMIL* excess body mass index (BMI) loss, *%TBWL* total body weight loss, *HDL* high-density lipoprotein cholesterol, *RYGB* Roux-en-Y gastric bypass, *SG* sleeve gastrectomy, *TC* total cholesterol, *TG* triglycerides, *T2DM* type 2 diabetes mellitus

### Weight loss

As the reduction of weight significantly influences the outcomes of MetS following bariatric and metabolic surgery, a growing body of research showcases its effectiveness in achieving excess weight loss.

The first extensive multicenter study with 10 year follow-up showed a maximum 71 ± 25% excess weight loss (%EWL) after SG within a median duration of 12 months after surgery [30]. After 10 years, 60.4% of patients still maintained a mean %EWL of 53 ± 25%, which corresponds to about a 26.3 ± 13.4% decrease in their total body weight.

A meta-analysis by Yang et al., including 1381 cases from 15 randomized controlled trials comparing RYGB and SG, demonstrated better weight loss 3 and 5 years post-surgery with a mean %EWL of 69.3% for RYGB and 59.1% for SG at 5 years. However, no significant differences were found between the two procedures within the first three years [31].

Neither the SM-BOSS [22] nor the SLEEVEPASS [21] trial was able to demonstrate significant differences in weight loss between SG and RYGB 5 years after surgery. In the SM-BOSS trial, excess BMI loss (%EBMIL) did not significantly differ, with SG at 61.1% compared to RYGB at 68.3%. Even though in the SLEEVEPASS trial, the estimated mean percentage of %EWL at 5 years was higher after RYGB with 57% compared to 49% after SG it did not meet criteria for inequivalence. However, when merged individual-patient data from these two randomized controlled trials (RCTs) were analyzed, %EBMIL was superior after RYGB compared to SG, with values of 62.7% and 55.5%, respectively [23]. Similarly, 5-year data from the STAMPEDE trial revealed that patients who underwent RYGB experienced a greater total body weight loss compared to those who had SG, with weight loss amounts of 23.2 ± 9.6 kg for RYGB and 18.6 ± 7.5 kg for SG [4].

### Arterial hypertension

Epidemiologic studies have demonstrated a direct correlation between the body mass index (BMI) of patients and both systolic and diastolic hypertension [32].

A systematic review involving 33 studies and 3997 patients revealed that hypertension was reduced in 75% of cases after SG, with complete resolution occurring in 58% after a follow-up period of approximately 16.9 ± 9.8 months [24].

Data from the SLEEVEPASS trial indicated that after 5 years, 29% (20/68) of patients in the SG group discontinued their antihypertensive medications, compared to 51% (37/73) in the RYGB group [21]. Additionally, 35% (24/68) of the SG group required fewer medications, while 30% (22/73) in the RYGB group did. However, no improvement was observed in 35% (24/68) of the SG group versus 19% (14/73) in the RYGB group.

At the 10 year follow-up, 8% (6/72) of patients after SG and 24% (16/68) after RYGB had stopped their medications [25]. Furthermore, 32% (23/72) in the SG group reduced their medication dosage, compared to 24% (16/68) in the RYGB group. Lastly, 60% (43/72) of the SG group and 53% (36/68) of the RYGB group reported no change in their medication usage.

Interestingly, Cavarretta et al. were able to demonstrate cardiac remodeling after SG using echocardiography [26]. The study showcased a decrease in left ventricular mass along with enhancements in diastolic function, demonstrating an overall improvement in cardiac function. These alterations appear to be associated with the weight loss induced by SG and the amelioration of MetS.

### Dyslipidemia

In terms of improvements in lipid profiles, research indicates positive outcomes following SG. Results are from the SleeveBypass trial, a phase III multicenter randomized controlled trial comparing SG and RYGB showed a significant improvement in dyslipidemia after 5 years in both groups, but RYGB showed greater efficacy. Specifically, 62.0% (44/71) of patients after SG experienced improvement, compared to 83.1% (54/65) after RYGB [27].

The SM-BOSS trial further supports these findings. Five years after surgery, complete remission of dyslipidemia was achieved in 42.6% (29/68) of patients in the SG group, compared to 62.3% (33/53) in the RYGB. Significant improvements were observed in both groups for total cholesterol, high-density lipoprotein cholesterol (HDL-C), low-density lipoprotein cholesterol (LDL-C), and triglycerides [22].

### Diabetes

STAMPEDE directly compared medical and bariatric surgical management of obesity and diabetes [28]. At 12 months, the primary outcome, defined as achieving a glycated hemoglobin level of 6.0% or less with or without the use of diabetes medications, was more than three times higher following SG or RYGB (42% and 37%, respectively) compared to medical therapy alone (12%). 5 year follow-up data revealed that patients who underwent surgical procedures experienced a greater mean percentage reduction from baseline in glycated hemoglobin levels compared to those who received only medical therapy alone (2.1% vs. 0.3%) [4]. The primary outcome was reached by 5% of those with medical therapy alone, compared to 29% who underwent RYGB and 23% who underwent SG.

A multicenter study conducted by the National Patient-Centered Clinical Research Network (PCORNet) revealed that the remission rates for Type 2 diabetes mellitus (T2DM) were 59.2% for patients who underwent RYGYB and 55.9% for those who had SG at one year post-surgery. These rates increased significantly over time, with 86.1% of RYGB patients and 83.5% of SG patients achieving remission at 5 years post-surgery [29]. Remission was defined as the first measurement post-surgery where HbA1c levels fell below 6.5% after at least 6 months (either before or after surgery) without prescriptions for T2DM medication.

## Safety

The relative technical simplicity, together with a low morbidity, remaining endoscopic access to the gastrointestinal tract, absence of anastomoses and minimal nutritional deficiencies, underlines SG’s safety as an established procedure [10].

In the SM-BOSS trial, early complications (0–30 days post-surgery) were observed in 0.9% following SG and 4.5% after RYGB [22]. Similarly, also late complications (from postoperative day 30 to 5 years) were slightly in favor of the SG group, in which 14.9% required further surgical or endoscopic interventions compared to 17.3% after RYGB.

Another study comparing the safety of SG and RYGB up to 2 years showed that patients undergoing SG experienced fewer re-interventions (9.9% vs 15.6%), fewer complications (6.6% vs 9.6% for RYGB) and lower overall healthcare costs ($47,891 vs $55,213) than those who had RYGB [33]. However, at the 2 year mark, revisions were slightly more frequent in SG patients (0.6% vs 0.4%).

A further comparative study revealed a lower cumulative incidence of mortality (4.3% vs 5.7%) at 5 years after SG, as well as less complications (22.1% vs 29.0%), and re-interventions (25.2% vs 33.6%) [34]. The adjusted hazard ratio of all-cause hospitalization and emergency department visits was lower for SG at 1 and 3 years after surgery, but similar between groups at 5 years.

When compared to OAGB, the overall complication rates were reported to be similar between the two groups. However, OAGB has a higher incidence of ulcers (7.7% vs. 1.7%) and nutritional deficiencies (20.0% vs. 12.0%), while SG shows a higher prevalence of suboptimal weight response (25.7% vs. 6.4%) and reflux (17.8% vs. 8.3%) [35].

## Sleeve gastrectomy—to do or not to do?

SG offers several advantages over other bariatric procedures, such as being technically easier and faster to perform, with fewer complications. It provides excellent weight loss and improves associated comorbidities, with the added benefit of being convertible to other procedures if necessary [8, 10].

However, despite these benefits, there are notable risks and challenges associated with SG. Long-term data indicate that a significant number of patients may experience suboptimal weight response or develop gastroesophageal reflux disease (GERD). De novo reflux can occur in up to 30% of patients, with persistent or worsened GERD symptoms in half of all patients after ten years [36]. Boza et al. reported de novo reflux in 26.7% of their cohort after 5 years [37].

Chronic reflux can lead to Barrett’s metaplasia, which is always a highly discussed topic when it comes to SG. While Genco et al. [38] showed newly diagnosed Barrett’s metaplasia in 17.2% of patients at an average follow-up of 4.8 years, a prospective clinical cohort study including a total of 169 patients with a median follow-up of 7 years showed no significant difference in the incidence of de novo Barrett esophagus (BE) (1.2% after SG vs 3.6% after RYGB) [39]. Similarly, the SLEEVEPASS trial showed a cumulative incidence of BE of 4% after SG and 4% after RYGB, which is markedly lower than in earlier trials and similar between both procedures [25]. However, theses variations might be due to varying definitions of BE regarding intestinal and gastric metaplasia and also the potential variability of endoscopic assessment of BE between countries [40].

However, issues like worsening of GERD or suboptimal weight response sometimes necessitate conversion to another procedure. Studies have shown that up to 36% of patients may require conversion within 10 years due to suboptimal weight loss and/or GERD [30].

## Conclusion

SG stands out as an important tool in the battle against the MetS and obesity. Its impact on metabolic health is proven by its ability to produce notable weight loss, improve the cardiac risk profile, and manage comorbidities linked with obesity, such as diabetes, hypertension, and dyslipidemia. However, long-term studies suggest that RYGB may offer more durable weight loss and greater effectiveness in resolving metabolic comorbidities over time. SG, on the other hand, has the advantage of fewer early complications and reduced nutritional deficiencies compared to RYGB.

Nevertheless, it’s important to recognize suboptimal weight response or long-term complications like GERD, which might necessitate conversion to a hypoabsorptive bariatric-metabolic procedure later on. Therefore, careful patient selection and a long-term, continuous follow-up are essential to ensure optimal patient outcomes, as obesity still remains a chronic disease.

## Data Availability

Data sharing not applicable to this article as no datasets were generated or analyzed during the current study.
